# Cross-sectional study of seropositivity, lung lesions and associated risk factors of the main pathogens of Porcine Respiratory Diseases Complex (PRDC) in Goiás, Brazil

**DOI:** 10.1186/s40813-019-0130-0

**Published:** 2019-10-14

**Authors:** José Vanderlei Burim Galdeano, Thaís Gasparini Baraldi, Maria Eugênia Silveira Ferraz, Henrique Meiroz de Souza Almeida, Marina Lopes Mechler-Dreibi, Willian Marcos Teixeira Costa, Hélio José Montassier, Luis Antonio Mathias, Luís Guilherme de Oliveira

**Affiliations:** 1 School of Agricultural and Veterinarian Sciences, São Paulo State University (UNESP), Jaboticabal, SP Brazil; 2 COMIGO – Cooperativa Agroindustrial dos Produtores Rurais do Sudoeste Goiano, Rio Verde, GO Brazil; 3 Ceva Saúde Animal, Paulínia, SP Brazil

**Keywords:** Mycoplasma-like lung lesion, Pneumonia, PRDC, Pleuritis, Serology, Swine

## Abstract

**Background:**

The objective of the study was to evaluate the occurrence and severity of Porcine Respiratory Diseases Complex (PRDC) pathogens in the Goiás State, Brazil. Were assessed the serological antibodies occurrency of *Mycoplasma hyopneumoniae*, *Actinobacillus pleuropneumoniae* and swine influenza virus (SIV), as well as the evaluation of pulmonary Mycoplasma-like lung lesions, pleuritis, histopathological lesions and diseases occurrence associated with risk factors, such as management, housing and productive indexes. We conveniently selected 2536 animals for serology testing, and 900 lungs at slaughtering of animals from 30 multisite herds in Goiás State, Brazil.

**Results:**

For *M. hyopneumoniae,* all herds presented seropositive animals at some stage of production. Even though most herds (29/30) vaccinated against this pathogen, 90.0% (27/30) of the herds presented at least 50.0% of seropositive animals in finishing and slaughter. Overall, antibodies against *A. pleuropneumoniae* were present in lower occurrence, varying from 22.4% of the animals in the nursery phase to 1.3% of the animals at slaughter. Conversely, SIV circulated in most herds, with 29 seropositive herds without vaccination. The occurrence of anti-SIV antibodies was higher at slaughter (74.5% of the animals) than nursery (41.8% of the animals), and at slaughter, 23 herds (76.7%) presented at least 50.0% of seropositive animals. All herds presented animals with pulmonary Mycoplasma-like lung lesions, and of the 900 lungs evaluated in the slaughterhouse, 665 (73.9%) presented an average Mycoplasma-like lung lesions of 7.3%. Evaluations of the pneumonia index (PI) showed that 73.3% of the herds were strongly affected by a pathology that manifested itself in different presentation forms. Microscopically, there was a predominance of bronchopneumonia lesions (74.6% of affected lungs), with a high occurrence of the chronic form (57.1%), and there was a moderate to marked proliferation of bronchial associated lymphoid tissue (BALT) in 64.1% of the affected lungs. Pleuritis were observed in 13.5% of the animals.

**Conclusion:**

Serological tests evidenced that antibodies against App and SIV were present in the Goiás State herds, and high occurrence of *M. hyopneumoniae* antibodies in finishing phases and slaughter may be influenced by pathogen circulation in vaccinated herds, leading to respiratory lesions at slaughter. Additionally, swine influenza virus was broadly disseminated in technified herds in Goiás State.

## Background

The Porcine Respiratory Disease Complex (PRDC) is still one of the most challenging health issues faced by the pig industry worldwide due to the interaction of viral and bacterial infectious pathogens, environmental conditions and management practices [1]. Several bacterial and viral pathogens are involved in respiratory diseases in pigs. *Mycoplasma hyopneumoniae* (*M. hyopneumoniae*) and *Actinobacillus pleuropneumoniae* (App) are considered the most important primary bacterial pathogens associated with pulmonary lesions [2], and swine influenza virus (SIV) is the most important primary viral pathogen in Brazil followed by porcine circovirus type 2 (PCV2), since porcine reproductive and respiratory syndrome virus (PRRSV) has never been reported [3].

In Brazil, the infections caused by *M. hyopneumoniae* are considered a primary cause of respiratory problems [4]. Bacteriological, histopathological and immunohistochemical tests on lung samples showed that 97.2% of pneumonia lesions were caused by *M. hyopneumoniae* and *Pasteurella multocida* co-infection, highlighting the importance of those pathogens interaction for swine respiratory disease [5]. The presence of pulmonary consolidations, although not pathognomonic of *M. hyopneumoniae* infections [1], is positively associated with seropositive herds. The characteristic lesions of pneumonia, lymphadenomegaly and pulmonary congestion are strongly related to positive PCR results for *M. hyopneumoniae* and *Pasteurella multocida* [6].

In different countries, respiratory lesions are the main reason for lung condemnation as well as a lower slaughter line pace that is attributed to the higher percentage of carcasses that need to be trimmed [7] and are responsible for approximately 50.0% of the rejections in this species [8]. Thus, the objective of this study was to evaluate the occurrence and severity of respiratory diseases in multisite swine herds in the state of Goiás, in Brazil, by studying the presence of antibodies against *M. hyopneumoniae*, App and SIV in different production phases. We also evaluated Mycoplasma-like lung lesions, pleuritis, histopathological lesions and diseases occurrence associated with risk factors, such as management, housing and productive indexes.

## Methods

### Study design

This study was conducted in 2016 and 2017. We selected by convenience 30 production units with multisite farrow-to-finish herds. Goiás State contains approximately 100.000 sows, and the herds sampled represent 51.0% of Goiás production. Out of the 30 herds, 24 contained nursery and breeding sites at the same farm. In the other six herds, the nursery was located in the same farms as the finishing animals. The average herd size of sampled units ranged from 400 to 4000 sows. Briefly, five herds had between 400 and 1000 sows, 12 herds had between 1001 and 2000 sows, and 13 herds had sow herd between 2001 and 4000 sows.

The number of animals to be sampled in each class of production site (nursery, growing, finishing and slaughter) was determined using the following formula [9].

$$N=\left[1-{\left(1-C\right)}^{\frac{1}{\left(D\times S\right)}}\right]\times \left[\frac{M-\left(D\times S-1\right)}{2}\right]$$

in which:

N = required number of samples or animals to be investigated;

C = degree of confidence of the sampling procedure;

D = number of units with disease/infection (estimated prevalence);

S = sensitivity of the diagnostic test;

M = number of units (animals) of the herd to be studied.

The expected occurrence of pneumonia in each of the classes (11% for nursery and growing phases, 35% for finishing phase) was based on studies previously conducted in the southern region of Brazil [10–12]. By using the formula, we considered the lowest diagnostic sensitivity (S) among the ELISA tests (IDEXX, Westbrook, ME, USA) to be 85% and the degree of confidence to be 95% [13–15]. Thus, the number of needed study animals (M) from the total population of the 30 herds was 15 nursery animals, 30 growing animals, 10 finishing animals and 30 slaughter age animals, totaling 85 animals per farm. Considering this was a cross-sectional study, blood samples of the farm animals were collected on the same day, and the slaughterhouse animals were collected a few days later.

Blood was collected by jugular vein puncture using disposable sterile needles and syringes, deposited in commercial tubes (BD® Franklin Lakes, New Jersey, USA) with clot activator, and centrifuged at 1500 x g for 10 min. The sera were separated, aliquoted and stored at − 20 °C until analysis. In order to standardize the age of the animal samples, the nursery pigs were sampled at 50 days-age, the growing pigs at 94 days-age, the finishing pigs at 130 days-age, and the slaughter pigs were sampled between 150 and 180 days-age. Vaccination scheme of all herds sampled is presented in Table 1. For ethical reasons, the name of each vaccine was changed for letters.

**Table 1:** Vaccination procedures adopted by each of the 30 herds sampled, followed by the timing of vaccine use, in the state of Goiás, Brazil (2016/ 2017)

Farm ID	*M. hyopneumoniae*	App	SIV
1	Vaccine A – 7d, 21d*	Vaccine D – 21d, 35d*	None
2	None	None	None
3	Vaccine B – 21d*	Vaccine E – 30d, 50d*	None
4	Vaccine B - 21d*	Vaccine E – 30d, 50d*	None
5	Vaccine B - 21d*	Vaccine E – 30d, 50d*	None
6	Vaccine A – 15d, 35d*	None	None
7	Vaccine C – 35d*	Vaccine F – 18d, 39d*	None
8	Vaccine A – 7d, 21d*	None	None
9	Vaccine A – 21d*	None	None
10 to 30	Vaccine C – 18d*	Vaccine F – 18d, 39d*	None

* *d* (days) - age of piglet at vaccination

In another approach, the slaughtering of the pigs from all previously sampled herds was monitored, and at least 30 animals were selected from each farm as commonly practiced in Brazil [16]. In the slaughterhouses, blood samples were collected at the time of bleeding, and the respective lungs of each pig was evaluated and classified according to the pneumonia index (PI). Mycoplasma-like lung lesions score was evaluated by the same person that no access to any epidemiological data of the herds. Lesion grading was evaluated according to the total area of pneumonia, using the mean of each lobe score in relation to the total lung area [16, 17]. Herds with average PI of up to 0.55 were considered free of pneumonia (Grade 0). Herds with average indexes between 0.56 and 0.89 obtained an intermediate classification (Grade 1), in which the presence of pneumonia occurred but did not characterize a threat to the herd. Herds with indexes above 0.90 were considered very affected, with severe occurrences of pneumonia in the herd (Grade 2) [16]. Regarding pleuritis, the lungs were evaluated for the presence or absence of lesions.

The mediastinic lymph nodes and lungs presenting Mycoplasma-like lung lesions in the pulmonary parenchyma were selected and collected for histopathological evaluation. For microscopic analysis, all tissue samples were fixed by immersion in formalin for 72 h, transferred to 70% alcohol, and then embedded in paraffin. Samples of tissues were cut and stained with hematoxylin/eosin (HE) in the CEDISA Laboratory (Center for Diagnosis of Animal Health, Concordia, Brazil).

A total of 2536 blood serum samples collected from herds and slaughterhouses were tested for the detection of specific antibodies against *M. hyopneumoniae*, App and SIV by the Enzyme Linked Immunosorbent Assay – ELISA with the commercial kits IDEXX (Westbrook, ME, USA) M. hyo. Ab Test, IDEXX APP-ApxIV Ab Test, and IDEXX Influenza A Ab test, respectively, according to manufacturer’s instructions. The relative level of antibody in the sample was determined by calculating the sample to positive (S/P) ratio, and the cut-off values for *M. hyopneumoniae,* App and SIV were 0.40, 0.50, and 0.40, respectively.

### Epidemiologic data collection

In every sampled farm, an epidemiologic questionnaire with 114 questions was used to collect data regarding: zootechnical indexes, management practices, application of biosecurity measures, which vaccines were used, the structure of animals facilities, adoption of welfare practices, environmental data and the feeding system used in the farm. Categorical data was obtained through dichotomous questions, which only allowed yes or no as an answer. Continuous data (mostly zootechnical indexes) were obtained by questions which allowed any type of numeric answer, The questionnaire information was filled by authors during an interview with farm owner and technical team. The interviewer was trained in order to not influence the farmer and the technical team answers. The variables are described in Additional file 1.

### Data analysis

#### Serological results

All values of frequencies obtained for each strata (age group) had a 95% confidence interval (CI) calculated using Wilson’s methodology.

#### Epidemiologic data

The categorical data obtained from the 30 sampled herds was further tabulated. The outcomes selected to analyze the explanatory variables were the serology results of each strata and the PI of the herds.

In order to investigate potential association between the outcomes and categorical explanatory variables, the Fisher’s Exact Test (*p* < 0.2) was used. All potential significant associations were then used in univariate logistic regression models (*p* < 0.05) to assess the association and to obtain the OR value and its 95% confidence interval (CI). For this analysis two explanatory variables were transformed into categorical data, regarding stocking density at weaning, more than 0.3m^2^/animal was attributed value 1, while less than 0.3m^2^/animal was given the value 0; regarding stocking density at growing and finishing, more than 1m^2^/animal was given the value 1 and less than 1m^2^∕animal was given the value 0. Another converted variable was the number of animals per trough at weaning, growing and finishing phase, more than 35 animals∕trough was given the value 1 and less than 35 animals∕trough was given the value 0. The data analysis was performed using Epi Info™ (Centers for Disease Control and Prevention, Atlanta, USA).

Association continuous variables investigated as potential risk factors and the serological data obtained (outcome), were detected by simple linear regression models were performed (*p* < 0.05) using the software R [18]. Whenever significant associations were found, the residuals were analyzed to verify whether the statistical assumptions of the simple linear regression were met. In cases in which the assumptions were not met, the association was investigated using Spearman’s coefficient (*p* < 0.05) calculation for non-parametric data.

## Results

### Serology

The serological results for the three infectious pathogens per class and the respective 95% CI are shown in Additional files 2, 3 and 4. Regarding *M. hyopneumoniae*, we were not able to differ vaccine antibodies from infection, which does limit the interpretation of the data. The serological data indicated that SIV probably circulated in most of the herds at all stages of production, with a higher proportion of positive samples at the later stages. Likewise, the data observed for App had a particular characteristic. Disregarding the detection of maternal antibodies in nursery, a low occurrence was observed in subsequent phases, which may indicate pathogen circulation in the herds (Table 2).

**Table 2:** Percentage of seropositivity of *M. hyopneumoniae,* App and SIV antibodies in the pigs in the nursery, growing, and finishing phases and at slaughter from the 30 sampled herds in the State of Goiás, Brazil (2016/2017). S/P cut-off values for *M. hyopneumoniae,* App and SIV were 0.4, 0.5, and 0.4, respectively

Seropositivity												
Herd ID	Nursery (50d^a^)			Growing (94d)			Finishing (130d)			Slaughter (150-180d)		
	Mhyo	App	SIV	Mhyo	App	SIV	Mhyo	App	SIV	Mhyo	App	SIV
1	66.7	13.3	66.7	86.7	10.0	6.7	100.0	10.0	0.0	100.0	5.0	0.0
2	0.0	0.0	13.3	0.0	0.0	57.1	10.0	0.0	0.0	0.0	0.0	3.3
3	78.6	0.0	92.9	83.3	0.0	83.3	93.3	0.0	100.0	93.3	0.0	100.0
4	40.0	0.0	86.7	46.7	25	63.3	100.0	0.0	60.0	93.3	3.3	30.0
5	80.0	0.0	0.0	63.3	0.0	0.0	10.0	0.0	0.0	73.3	0.0	3.3
6	93.3	0.0	66.7	66.7	0.0	100.0	90.0	0.0	50.0	93.3	0.0	96.7
7	20.0	26.7	60.0	10.0	0.0	96.7	20.0	0.0	100.0	100.0	10.0	96.7
8	100.0	28.6	92.9	96.3	0.0	22.2	100.0	0.0	40.0	46.7	0.0	76.7
9	0.0	69.2	0.0	18.5	0.0	0.0	100.0	0.0	0.0	80.0	6.7	0.0
10	6.7	25.0	46.7	10.0	5.0	70.0	70.0	4.6	30.0	93.3	0.0	100.0
11	0.0	0.0	13.3	20.0	0.0	86.7	50.0	0.0	45.5	100.0	0.0	46.7
12	0.0	6.7	57.1	0.0	0.0	40.0	90.0	10.0	100.0	100.0	0.0	100.0
13	6.7	13.3	73.3	26.7	0.0	56.7	70.0	0.0	100.0	100.0	0.0	100.0
14	6.3	0.0	26.7	3.3	0.0	90.0	40.0	0.0	70.0	43.3	6.67	76.7
15	13.3	20.0	40.0	13.3	0.0	96.7	30.0	10.0	80.0	93.1	10.0	93.3
16	0.0	20.0	13.3	16.7	0.0	100.0	20.0	0.0	100.0	96.7	0.0	93.3
17	0.0	13.3	40.0	10.0	0.0	100.0	100.0	0.0	100.0	100.0	0.0	93.3
18	40.0	80.0	13.3	3.3	0.0	93.3	30.0	0.0	100.0	100.0	3.3	100.0
19	6.7	0.0	46.7	20.0	0.0	86.7	70.0	0.0	60.0	100.0	6.7	80.0
20	0.0	6.7	13.3	3.3	0.0	6.7	20.0	0.0	50.0	86.7	43.3	78.6
21	46.7	20.0	13.3	16.7	0.0	3.3	20.0	0.0	100.0	83.3	0.0	83.3
22	0.0	60.0	20.0	3.3	0.0	100.0	60.0	0.0	100.0	96.7	10.0	96.7
23	0.0	46.7	53.3	16.7	0.0	96.7	100.0	0.0	100.0	100.0	0.0	100.0
24	26.7	13.3	66.7	23.3	0.0	96.7	100.0	0.0	100.0	100.0	0.0	100.0
25	0.0	6.7	33.3	0.0	0.0	100.0	10.0	10.0	50.0	100.0	16.7	96.7
26	6.7	33.3	26.7	10.0	3.3	96.7	100.0	0.0	100.0	100.0	20.0	66.7
27	13.3	18.8	46.7	3.3	0.0	100.0	40.0	0.0	60.0	100.0	13.3	83.3
28	20.0	26.7	26.7	16.7	0.0	100.0	47.4	0.0	10.0	100.0	0.0	3.3
29	6.7	33.3	60.0	40.0	0.0	86.7	10.0	0.0	80.0	100.0	10.0	100.0
30	0.0	40.0	46.7	0.0	3.3	46.7	100.0	0.0	100.0	100.0	0.0	83.3
X	22.4	20.5	41.8	24.2	1.3	69.8	61.7	1.6	66.1	88.4	5.7	74.5

^a^*Mhyo Mycoplasma hyopneumoniae*, *App Actinobacillus pleuropneumoniae*, *SIV* Swine influenza virus. *d* days; age of pigs

#### Serology for mycoplasma hyopneumoniae

Regarding *M. hyopneumoniae*, all herds presented seropositive pigs at some stage of production, but most of the herds were vaccinated against *M. hyopneumoniae*, excluding farm 2, which was certified free of *M. hyopneumoniae* without vaccination. Regarding the nursery piglets, 11 herds presented seronegative animals (2, 9, 11, 12, 16, 17, 20, 22, 23, 25 and 30) (Table 2). Four of them presented seronegative animals during growing phase, and seropositive animals in the finishing phase (2, 12, 25 and 30), while the other seven herds presented seropositive animals in the growing and finishing phases (9, 11, 16, 17, 20, 22 and 23) (Table 2). At slaughter, only farm 2 showed seronegative animals. This farm presented 10.0% of seropositive animals in the finishing phase, being negative in all other previous phases and at slaughter. Only five herds (1, 3, 5, 6 and 8) presented more than 50.0% of seropositive pigs in both the nursery and growing phases. At finishing phase, 17 herds presented more than 50.0% of seropositive pigs (Table 2). At slaughter, 27 herds presented more than 50.0% of seropositive pigs, with 23 herds presenting more than 90.0%.

#### Serology for APP

Regarding App, 25 herds were seropositive in at least one of the production phases, and only five were seronegative in all of them (2, 3, 5, 6, 11). Considering that most of the herds were vaccinated in the nursery phase (excluding herds 2, 6, 8 and 9), we observed only 22 herds with seropositive animals in this phase. In the growing phase, only four herds were positive, and one of them was negative in the nursery phase. Five herds presented seropositive pigs at the finishing phase, and at slaughter, 14. Farm 1 presented seropositive animals at all stages of production.

Only three herds had more than 50.0% of seropositive animals in the nursery phase (9, 18 and 22) (Table 2). The occurrences varied from 22.4% in the nursery phase to 1.3% in the growing phase. This low occurrence was also observed at slaughter.

#### Serology for SIV

Regarding the serology for SIV, Farm 9 was the only seronegative. The number of seropositive animals was high in the later production phases in eight herds (2, 4, 5, 9, 11, 20, 26 and 28) (Table 2) and low in five herds (1, 2, 4, 8 and 28). Farm 5 presented seropositive animals only at slaughter. Since vaccination against SIV was not practiced, the number of seropositive animals from nursery to slaughter ranged from 41.8 to 74.5%. At slaughter, 24 herds had at least 50% seropositive animals for SIV.

### Pulmonary evaluation at slaughter

All herds presented animals with Mycoplasma-like lung lesions at slaughter. In individual evaluations, 24.4% (220/900) of the animals presented lung lesion score higher than 10%. In general, the mean percentages of lung lesions ranged from 0.2 to 17.4%, with an average of 7.3%. All herds contained animals with Mycoplasma-like lung lesions, including Farm 2 (Table 3).

**Table 3:** Results of the pulmonary evaluations of 900 pigs at slaughter related to the percentage of Mycoplasma-like lung lesions areas and pleuritis, as well as the classification of the herds evaluated for PI. The animals were from commercial herds in the state of Goiás, Brazil (2016/ 2017)

Farm	% of average lung lesion score	PI	Classification of the herd by PI^a^	% of pleuritis	% of animals with lung lesions
01	7.9	1.13	2	10.0	86.7
02	0.2	0.07	0	0.0	6.7
03	5.8	1.0	2	6.7	86.7
04	5.0	0.80	1	6.7	56.7
05	4.0	0.83	1	3.3	73.3
06	6.6	1.10	2	10.0	80.0
07	11.4	1.50	2	10.0	90.0
08	14.5	1.87	2	13.3	96.7
09	3.3	0.73	1	13.3	70.0
10	3.9	0.90	2	13.3	76.7
11	3.7	0.87	1	13.3	80.0
12	7.2	1.17	2	23.3	83.3
13	9.7	1.43	2	10.0	93.3
14	3.8	0.87	1	3.3	73.3
15	5.7	1.03	2	6.7	86.7
16	7.5	1.23	2	6.7	86.7
17	6.0	1.03	2	3.3	80.0
18	7.5	1.10	2	50.0	80.0
19	12.0	1.72	2	6.7	96.7
20	7.5	1.20	2	10.0	86.7
21	7.5	1.30	2	6.7	83.3
22	6.6	1.10	2	6.7	73.3
23	3.7	0.90	2	6.7	80.0
24	17.4	2.03	2	13.3	96.7
25	11.4	1.50	2	6.67	93.3
26	9.3	1.40	2	13.3	93.3
27	4.0	0.90	2	13.3	76.7
28	14.9	1.93	2	13.3	96.7
29	5.3	0.87	1	3.3	66.7
30	5.6	0.97	2	6.7	80.0
Average	7.3	1.15		10.3	80.3

*Herds with pneumonia indexes of up to 0.55 were considered free of pneumonia (Grade 0). Herds with indexes between 0.56 and 0.89 obtained an intermediate classification (Grade 1), in which the presence of pneumonia occurred but did not characterize a threat to the herd. Herds with indexes above 0.90 were considered with severe occurrences of pneumonia in the herds (Grade 2)

For the pneumonia index, only one farm was classified as grade 0, six as grade 1, and 23 as grade 2, the highest score in this scoring system. The herds with the worst score represented 76.7% of the herds (Table 3). With regard to pleuritis, 29 herds had at least one affected animal, with occurrences varying from 3.3 to 50.0% and an average of 10.3% (Table 3). The farm with the highest percentage of pleuritis (farm 18) was negative for ApxIV in the growing and finishing stages and presented antibodies against the toxin at slaughter. The occurrence of animals with Mycoplasma-like lung lesions ranged from 6.7 to 96.7%, with a mean of 80.3%. In addition, we observed eight lungs with abscess injuries at slaughter.

### Histopathology

One lung lesion fragment was collected per pig at slaughter. In total, 665 fragments were analyzed and 15 different pathological pictures were observed (Table 4). There was a predominance of bronchopneumonia (74.6%; 496/665), with the chronic form being the most prevalent (57.1%. 380/665). In relation to pleuritis, the occurrence was 13.5% (90/665). Regarding bronchial associated lymphoid tissue (BALT), there were 63.8% of pathological findings (424/665), most of which were considered to be marked to moderate (64.2%; 272/665).

**Table 4:** Characterization of the different histological lesions observed in 665 lungs of pigs from commercial herds located in the state of Goiás, Brazil affected by Mycoplasma-like lung lesions at slaughter (2016/2017)

Histological features	Percentage of affected lung fragments
Chronic bronchopneumonia^a^	57.1% (380/665)
Suppurative bronchopneumonia	15.6% (104/665)
Granulomatous bronchopneumonia	0.3% (2/665)
Fibrinous bronchopneumonia^a^	0.2% (1/665)
Subacute bronchopneumonia	0.3% (2/665)
Catarrhal bronchopneumonia^a^	1.1% (7/665)
Chronic bronchiolitis^a^	3.3% (22/665)
Necrotic bronchiolitis^a^	10.5% (07/665)
Chronic pleuritis^a^	13.1% (87/665)
Fibrinous pleuritis	0.5% (3/665)
Pulmonary abscess	0.1% (1/665)
Interstitial pneumonia	3.6% (24/665)
Bronchointerstitial pneumonia^a^	4.5% (30/665)
Granulomatous pneumonia	0.3% (2/665)
Chronic pneumonia^a^	0.3% (2/665)
Bronchial associated lymphoid tissue (BALT)	Percentage of affected lungs
Absence of hyperplasia	36.2% (241/665)
Mild hyperplasia	27.1% (115/424)
Discrete hyperplasia	8.7% (37/424)
Moderate hyperplasia	35.4% (150/424)
Total	63.8% (424/665)

^a^Some lungs presented more than one type of histopathological finding but were classified according to the most extensive/predominant lesion

### Risk factor association

The logistic regression of the categorical variables showed potential associations between the investigated variables and the occurrence of APP, M. hyopneumoniae and SIV. APP seropositivity at slaughter was positively associated with more than 30 pigs per through (OR: 12.24; 95%CI 1.27–118.35), while using protocols of empty period between batches in the growing-finishing pig facilities was labeled as a protective factor (OR: 0.10; 95%CI 0.02–0.66).

The variable “Cross-fostering” in the maternity facilities, which aimed at redistributing pigs to standardize the size of the litters, was inversely associated with the SIV seropositivity in the growing-finishing pigs (OR: 0.92; 95%CI 0.85–0.99). The OR and *p* values of all associations are shown in Table 5. A logistic regression model was attempted including all variables with significant association in the univariate analysis; however, no significant results were obtained.

**Table 5:** OR and *p-*values of the associations found by univariate logistic regression between the investigated variables and the SIV, APP and *M. hyopneumoniae* seropositivity in the sampled herds

Dependent variable	Independent variable	*P*	OR	95% CI
App weaning	Colostrum ingestion aid	0.03	1.04	1.00–1.09
App growing-finishing	Empty period between batches in maternity facilities	0.02	0.10	0.02–0.66
App slaughter	More than 30 pigs per through in growing-finishing phases	0.03	12.24	1.27–118.35
SIV growing-finishing	Cross-fostering	0.04	0.92	0.85–0.99

### Linear regression

The continuous variables were submitted to a linear regression model in order to detect potential associations. Table 6 shows the R^2^ and *p* values obtained for each model.

**Table 6:** Associations detected between the APP, SIV and *M. hyopneumoniae* seropositivity and the investigated variables using univariate linear regression models (p < 0.05)

*Dependent variables*	*Independent variables*	R^2^	*p*
Average daily weight gain (g/day)	*M. hyopneumoniae* seropositivity at weaning	0.39	2.00 e-04
Average daily weight gain (g/day)	*M. hyopneumoniae* seropositivity at growing-finishing	0.34	7.00 e-04
Mortality in finishing phase (%)	SIV seropositivity in growing-finishing	0.38	2.00 e-04
Mortality in finishing phase (%)	Average pulmonary lesion extension (%)	0.17	2.00 e-02
Mortality in finishing phase (%)	PI	0.20	1.00 e-02
*M. hyopneumoniae* seropositivity in growing-finishing	Empty period between batches in growing-finishing facilities	0.26	4.00 e-03

The presence of *M. hyopneumoniae* antibodies at weaning was also inversely associated with ADWG. At the growing-finishing phase, it was also inversely associated with the number of days of empty period between batches and the adoption of cleaning and disinfection protocols at the finishing-growing facilities (Table 7). A positive association was detected between the *M. hyopneumoniae* seropositivity and the presence of other nearby pig herds.

**Table 7:** Associations obtained between the *Actinobacillus pleuropneumoniae*, *Mycoplasma hyopneumoniae* and influenza seropositivities in the sampled herds from Goiás, Brazil, when using Spearman’s coefficient test at *p < 0.05

*Dependent variable*	*Independent variable*	Coefficient	*p*
Average pulmonary lesion area	*M. hyopneumoniae* seropositivity at slaughter	0.38	0.035
Pneumonia index (PI)	*M. hyopneumoniae* seropositivity at slaughter	0.37	0.041
ApxIV seropositivity in growing animals	Well conserved barn curtains	−0.48	0.007
ApxIV herd level seropositivity	Performing “all-in/all-out” in the maternity facilities	−0.39	0.031
*M. hyopneumoniae* seropositivity in growing animals	Protocol for cleaning and disinfecting in growing/finishing phases	−0.39	0.034
*M. hyopneumoniae* seropositivity in growing animals	Performing “all-in/all-out” in the growing and finishing facilities	−0.46	0.011
*M. hyopneumoniae* seropositivity at slaughter	Other swine herds nearby	0.40	0.028
SIV seropositivity in growing animals	Other swine herds nearby	0.42	0.020

The SIV seropositivity in the growing/finishing phases was positively associated with mortality in the phase. Since the mortality in the finishing pigs also presented a positive association with higher PI and medium size pulmonary lesions, all aforementioned variables were used to create a multiple linear regression model (R^2^ = 0.43 and *p* < 0.05). Despite the relatively low R^2^, the model was significant, indicating a potential association between the variables.

Both PI and average pulmonary lesion size presented a positive and significant association with the *M. hyopneumoniae* seropositivity in the finishers when using the Spearman correlation coefficient (Table 7). Regarding App, an inverse correlation was observed between the seropositivity in the growing phase and the use of well-conserved barn curtains, and between the herd seropositivity and the use of “all-in/all-out” in the farrowing facilities.

## Discussion

A high seropositivity of *M. hyopneumoniae* antibodies was found at the finishing phase and slaughter (61.7 and 88.4% of the animals, respectively), although 13 herds had less than 50.0% of seropositive animals in finishing and at slaughter phases. Lower occurrence of antibodies were observed in nursery and growing animals (22.4 and 24.2%, respectively).

Taking into account the presence of Mycoplasma-like lung lesions at slaughter and the high occurrence of seropositive animals in finishing phases, it is possible to infer that vaccination was not preventing pathogen circulation in later phases in these herds. In addition, consequently, the high number of seropositive could have been be influenced by new infection. In an experimental study, *Mycoplasma hyopneumoniae* shedding did not show significant differences between vaccinated and non-vaccinated populations [19]. Thus, it is possible that pathogen circulation was still happening in finishing phases of the sampled herds, even though the majority of the herds were vaccinated for *M. hyopneumoniae*. However, since this study was cross-sectional and did not provide direct evidence of pathogen circulation, it is not possible to confirm that hypothesis.

The serological results for App showed a low occurrence of the ApxIV toxin antibodies, with the highest occurrence found in the nursery animals (20.5%), followed by 5.7% at slaughter, and the lowest in the growing and finishing animals (1.3 and 1.6%, respectively). Unlike the *M. hyopneumoniae* and SIV ELISA tests, the serological test used in this study did not detect vaccinal antibodies, and detected both pathogenic and non-pathogenic strains. The prevalence found in the nurseries may be explained by maternal derived antibodies, which are detectable until up to 12 weeks of age [20, 21].

Despite the non-vaccination against SIV, a high seropositivity in herds was observed along all stages of production (41.8% in the nursery phase, 69.8% in the growing phase, 66.1% in the finishing phase, and 74.5% at slaughter), a fact that was expected since enzootic SIV circulation in Brazilian herds has been reported [10]. Considering that the herds sampled corresponds to 51.0% of the Goiás State herd, the high seropositivity of SIV in non-vaccinated animals indicate that this pathogen is circulating in these herds, although there is no information about the strains. This result can be justified by animal production at multisite, since influenza A is usually introduced by animal transit [22].

To evaluate the degree of the occurrence of pneumonia in the herds, the PI of each farm was calculated. The mean value found among the 30 herds was 1.15, ranging from 0.07 to 2.03, confirming the presence of pneumonia in the herds. Considering this information, the state of Goiás appears to be in accordance with the prevalence data from other Brazilian states, with values varying from 0.86 to 1.54 and a mean of 1.03 [21]. Herds with PIs above 0.90 represent a serious occurrence of pneumonia, and the severity of the clinical status is proportional to the increase in the PI value [16]. The farm that presented the highest PI was also the one with the highest percentage of Mycoplasma-like lung lesions at slaughter (farm 24), with 96.7% (29/30) of the herd having this injury. In this farm, the percentage of seropositive animals for *M. hyopneumoniae* and SIV was higher in the latter phases than in the first ones, presenting 100% of seropositive pigs at slaughter. It is evident that there are herd risk factors that favor the occurrence of pneumonia and, if uncorrected, may intensify losses, mainly due to the association with secondary pathogens [11].

This fact leads us to believe that the occurrence of pleuritis was strongly associated with other pathogens. As observed in studies in slaughterhouses, 33.3% of the rejected carcasses presented pleuritis lesions [23]. The only farm with a PI of 0 also had no pleuritis lesions at slaughter. Despite the major negative serological results for *M. hyopneumoniae*, the farm presented animals seropositive to SIV in the nursery and growing phases.

Most of the histological lesions observed in this study (74.6%) were described as bronchopneumonia prevailing in the chronic form (57.1%), with characteristics of BALT hyperplasia ranging from moderate to severe (64.2%). These findings were suggestive, but not exclusive, of an *M. hyopneumoniae* infection since other respiratory pathogens may lead to similar lesions [24]. Other pathogens, such as SIV or *P. multocida*, should be considered within the most likely differential diagnoses [25]. The occurrence of high percentage of Mycoplasma-like lung lesions in the slaughterhouses suggests that vaccination protocols do not completely prevent this type of lesion, which reinforces the importance of management and biosecurity practices.

The injuries characterized as interstitial pneumonia (8.1%) bronchiolitis either chronic or necrotizing (4.4%), are generally induced by viral infection. However, animals in remission may present pulmonary lesions similar to those caused by *M. hyopneumoniae*, and additional confirmatory tests, such as PCR, are required [26].

The microscopic observation of chronic and fibrinous pleuritis (13.5%), the macroscopic observation of pleuritis (mean of 10.3%), and the occurrence of eight individual lung samples with abscesses are compatible with App infection. These data corroborate a previous study [23] in which only 8.0% of the animals presented lesions characteristic of pleuritis. On the contrary, there was no correlation with the presence of pneumonia in other parts of the lung. Studies confirm that the type and severity of the lung lesions in slaughter pigs result from dynamic and complex interactions between multiple infectious pathogens, such as co-infection of *M. hyopneumoniae* and *Pasteurella multocida*, mainly associated with pneumonia, and co-infection of App and *P. multocida*, mainly associated with pleuritis [5].

Regarding APP, a positive association was discovered between anti-ApxIV antibodies and more than 30 animals per trough (OR: 12.24). App transmission can occur by the air or by direct contact, specifically nose-to-nose contact. A previous study showed that nose-to-nose transmission was up to 10 times more frequent than air transmission [27]. Higher number of animals at trough feeding may be a reflection of the high density of animals per pen, which increases the direct contact between animals, consequently facilitating App transmission.

Protective factors were also detected in this study (inverse associations), such as having protocols of empty period between batches in the facilities and the App seropositivity in growers (OR: 0.10). Not performing an empty period between batches was positively associated with pleuritis and APP seropositivity in the swine herds, reinforcing the idea that all-in/all-out management is an important procedure in the prevention of respiratory diseases [2].

The linear regression detected an inverse association between the number of days of empty period between batches and the occurrence of *M. hyopneumoniae* antibodies in the growers. This association could be explained by the production flow of the sampled herds since most of the herds were multisite and the arrival of susceptible animals from the maternity sites was constant.

A reduction in the daily weight gain is the main production impact caused by *M. hyopneumoniae* in the pork producing chain, corroborating the inverse association found in this study between the ADWG and *M. hyopneumoniae* antibodies found in the growers and finishers. A previous study showed that losses due to *M. hyopneumoniae* infection could reach US$2.80 for each infected animal with pulmonary lesions [28].

Regarding SIV, the adoption of cross-fostering practices improve colostrum ingestion, resulting in a more robust protection against infectious diseases [29].

## Conclusion

This study showed that the seropositivity against *M. hyopneumoniae*, *Actinobacillus pleuropneumoniae* and SIV, and lung lesions suggest that PRDC could be a problem in the pig production systems in the state of Goiás. Thus, associated risk and protective factors were related with management, reinforcing the importance of good management practices for pig health.

## Supplementary information

**Additional file 1.** Description of the occurrence of the variables investigated as risk factors and their respective frequencies in the herds sampled in the state of Goiás, Brazil.

**Additional file 2. ***Mycoplasma hyopneumoniae* seroprevalence in the weaners, growers, finishers and pigs at slaughter from the 30 sampled herds from the state of Goiás, Brazil, and the respective 95% confidence interval (CI 95%).

**Additional file 3. ***Actinobacillus pleuropneumoniae* seroprevalence in the weaners, growers, finishers and pigs at slaughter from the 30 sampled herds from the state of Goiás, Brazil, and the respective 95% confidence interval (CI 95%).

**Additional file 4.** SIV seroprevalence in the weaners, growers, finishers and pigs at slaughter from the 30 sampled herds from the state of Goiás, Brazil, and the respective 95% confidence interval (CI 95%).

## Data Availability

Data sets used and analysed during the current study are available from the corresponding author upon reasonable request.
