# Supplementary material for: Cross-sectional study of seropositivity, lung lesions and associated risk factors of the main pathogens of Porcine Respiratory Diseases Complex (PRDC) in Goiás, Brazil
Source: Porcine Health Manag. 2019 Oct 14;5:23. doi: 10.1186/s40813-019-0130-0 (PMC6791015; doi:10.1186/s40813-019-0130-0)
Supplement: Supplementary file 1 — Additional file 1. Description of the occurrence of the variables investigated as risk factors and their respective frequencies in the herds sampled in the state of Goiás, Brazil. [file 40813_2019_130_MOESM1_ESM.docx]

**Supplementary Document IV.** Description of the occurrence of the variables investigated as risk factors and their respective frequencies in the herds sampled in the state of Goiás, Brazil.

| Variables | Variable  present | Variable  absent | Frequency  (%) |
| --- | --- | --- | --- |
| Leaking pen wall in nursery | 30 | 0 | 100.00 |
| Leaking pen wall in finishing and growing facilities | 23 | 7 | 76.67 |
| Leaking floor in maternity pens | 22 | 8 | 73.33 |
| Leaking floor in nursery pens | 29 | 1 | 96.67 |
| Compact floor in growing-finishing facilities | 23 | 7 | 76.66 |
| Water back pond | 19 | 11 | 63.33 |
| Presence of a hospital pen in the nursery | 15 | 15 | 50.00 |
| Presence of a hospital pen in the growing-finishing facilities | 25 | 05 | 83.33 |
| Ventilation and nebulization | 26 | 04 | 86.66 |
| Curtains in the nursery facilities | 30 | 00 | 100.00 |
| Curtains in good condition | 29 | 01 | 96.66 |
| Thermometer in the facilities | 27 | 03 | 90.00 |
| Temperature record and report | 03 | 27 | 10.00 |
| Dry feed in the nursery facilities | 30 | 00 | 100.00 |
| Dry feed in the growing-finishing facilities | 22 | 08 | 73.33 |
| Antibiotics in the feed | 29 | 01 | 96.66 |
| Daily pen cleaning | 26 | 04 | 86.66 |
| Adoption of cleaning and disinfection protocols in the nursery facilities | 29 | 01 | 96.66 |
| Adoption of cleaning and disinfection protocols in the growing and finishing facilities | 28 | 02 | 93.33 |
| Use of cold water for cleaning | 30 | 00 | 100.00 |
| Use of warm water for cleaning | 00 | 30 | 00.00 |
| Use of detergents for cleaning and disinfection | 30 | 00 | 100.00 |
| Use of protocol for detergent dilution | 30 | 00 | 100.00 |
| Use of disinfectant for cleaning | 30 | 00 | 100.00 |
| Use of a disinfectant dilution protocol | 30 | 00 | 100.00 |
| “All-in/all-out” performance in nursery | 28 | 02 | 93.33 |
| “All-in/all-out” performance in growing and finishing facilities | 24 | 06 | 80.00 |
| Empty-period in the maternity facilitates | 16 | 14 | 53.33 |
| Empty-period in the nursery facilities | 26 | 04 | 86.66 |
| Empty-period in the growing and finishing facilities | 24 | 06 | 80.00 |
| External aisle in the nursery facilities | 29 | 01 | 96.66 |
| External aisle in the growing and finishing facilities | 29 | 01 | 96.66 |
| Presence of other pig farms nearby | 22 | 08 | 73.33 |
| Mandatory shower for workers at the entrance | 30 | 00 | 100.00 |
| Clothes change required to enter the farm | 30 | 00 | 100.00 |
| Farm surrounded by fences | 30 | 00 | 100.00 |
| Swine boarding dock outside the farm | 30 | 00 | 100.00 |
| Feed trucks entering the farm | 00 | 30 | 00.00 |
| More than one genetic supplier | 03 | 27 | 10.00 |
| Empty-period adoption | 01 | 29 | 3.33 |
| Colostrum ingestion | 22 | 08 | 73.33 |
| Colostrum intake aid | 30 | 00 | 100.00 |
| Litter uniformization | 28 | 02 | 93.33 |
| Use of drying powder in the newborns | 29 | 01 | 96.66 |
| Preventive medication application at weaning | 08 | 22 | 26.66 |
| Use of creep for the maternity piglets | 29 | 01 | 96.66 |
| Creep with electric heating | 30 | 00 | 100.00 |
| Substrate in the creep floor | 16 | 14 | 53.33 |
| Substrate change | 15 | 15 | 50.00 |
| Thermostat in the creep | 13 | 17 | 43.33 |
| Differential heating for small piglets | 19 | 11 | 63.33 |
| Respiratory clinical signs in the maternity piglets | 1 | 29 | 3.33 |
| Respiratory clinical signs at the nursery facilities | 26 | 4 | 86.67 |
| Respiratory clinical signs at the growing and finishing facilities | 30 | 0 | 100.00 |
| Periodic veterinary assistance | 29 | 1 | 96.67 |
| Training course for new employees | 29 | 1 | 96.67 |
| Training course for substitute employees | 29 | 1 | 96.67 |
| Use of an APP vaccine | 26 | 4 | 86.67 |
| Use of a *Mycoplasma hyopneumoniae* vaccine | 29 | 1 | 96.67 |
| Use of a swine influenza vaccine | 0 | 30 | 0.00 |
| Less than 35 animals per trough in the nursery pens | 14 | 16 | 46.67 |
| Less than 35 animals per trough in the growing pens | 15 | 15 | 50.00 |
| Less than 35 animals per trough in the finishing pens | 14 | 16 | 46.67 |
| Liquid feed during the growing phase | 9 | 21 | 30.00 |
| Liquid feed at slaughter | 8 | 22 | 26.67 |
| Density lower than or equal to 0.3 m²∕animal in the nursery facilities | 20 | 10 | 66.67 |
| Density lower than or equal to 1 m² ∕animal in the growing facilities | 27 | 3 | 90.00 |
| Density lower than or equal to 1 m² ∕animal in the finishing facilities | 24 | 6 | 80.00 |
