# Supplementary material for: Cross-sectional study of seropositivity, lung lesions and associated risk factors of the main pathogens of Porcine Respiratory Diseases Complex (PRDC) in Goiás, Brazil
Source: Porcine Health Manag. 2019 Oct 14;5:23. doi: 10.1186/s40813-019-0130-0 (PMC6791015; doi:10.1186/s40813-019-0130-0)
Supplement: Supplementary file 2 — Additional file 2. Mycoplasma hyopneumoniae seroprevalence in the weaners, growers, finishers and pigs at slaughter from the 30 sampled herds from the state of Goiás, Brazil, and the respective 95% confidence interval (CI 95%). [file 40813_2019_130_MOESM2_ESM.docx]

**Supplementary Document II.** *Mycoplasma hyopneumoniae* seroprevalence in the weaners, growers, finishers and pigs at slaughter from the 30 sampled herds from the state of Goiás, Brazil, and the respective 95% confidence interval (CI 95%).

| **MHP** | **Nursery (n=15)** | | **Growing (n=30)** | | **Finishing (n=10)** | | **Slaughter (n=30)** | |
| --- | --- | --- | --- | --- | --- | --- | --- | --- |
| **Herd ID** | **Prevalence**  **(%)** | **CI 95%** | **Prevalence (%)** | **CI 95%** | **Prevalence**  **(%)** | **CI 95%** | **Prevalence (%)** | **CI 95%** |
| 1 | 66.67a | 41.72-84.83 | 86.67a | 70.32-94.69 | 100a | 72.25-100.00 | 100a | 88.65-100.00 |
| 2 | 0a | 0.00-20.39 | 0a | 0.00-11.35 | 10a | 1.79-40.42 | 0a | 0.00-11.35 |
| 3 | 78.57a | 53.32-92.17 | 83.33a | 66.43-92.66 | 93.33a | 63.49-99.12 | 93.33a | 78.67-98.15 |
| 4 | 40a | 19.82-64.25 | 46.67a | 30.24-63.86 | 100b | 72.25-100.00 | 93.33b | 78.67-98.15 |
| 5 | 80a | 54.81-92.95 | 63.33a | 45.51-78.12 | 10b | 1.79-40.42 | 73.33a | 55.55-85.82 |
| 6 | 93.33a | 70.18-98.81 | 66.67a | 48.78-80.77 | 90a | 59.58-98.21 | 93.33a | 78.67-98.15 |
| 7 | 20a | 7.05-45.19 | 10a | 3.46-25.62 | 20a | 5.67-50.98 | 100b | 88.65-100.00 |
| 8 | 100a | 79.61-100.00 | 96.3a | 82.79-99.29 | 100a | 72.25-100.00 | 46.67b | 30.24-63.86 |
| 9 | 0a | 0.00-20.39 | 18.52a | 8.53-35.66 | 100b | 72.25-100.00 | 80b | 62.69-90.50 |
| 10 | 6.67a | 1.19-29.82 | 10a | 3.46-25.62 | 70b | 39.68-89.22 | 93.33b | 78.67-98.15 |
| 11 | 0a | 0.00-20.39 | 20a | 9.50-37.31 | 50a | 23.66-76.34 | 100b | 88.65-100.00 |
| 12 | 0a | 0.00-20.39 | 0a | 0.00-11.35 | 90b | 59.58-98.21 | 100b | 88.65-100.00 |
| 13 | 6.67a | 1.19-29.82 | 26.67ab | 14.18-44.45 | 70b | 39.68-89.22 | 100b | 88.65-100.00 |
| 14 | 6.25abc | 1.06-29.28 | 3.33a | 0.59-16.67 | 40bc | 16.82-68.73 | 43.33c | 27.37-60.80 |
| 15 | 13.33a | 3.73-37.88 | 13.33a | 5.31-29.68 | 30a | 10.78-60.32 | 93.1b | 78.37-98.05 |
| 16 | 0a | 0.00-20.39 | 16.67a | 7.34-33.57 | 20a | 5.67-50.98 | 96.67b | 83.33-99.41 |
| 17 | 0a | 0.00-20.39 | 10a | 3.46-25.62 | 100b | 72.25-100.00 | 100b | 88.65-100.00 |
| 18 | 40a | 19.82-64.25 | 3.33b | 0.59-16.67 | 30ab | 10.78-60.32 | 100c | 88.65-100.00 |
| 19 | 6.67a | 1.19-29.82 | 20a | 9.50-37.31 | 70bc | 39.68-89.22 | 100c | 88.65-100.00 |
| 20 | 0a | 0.00-20.39 | 3.33a | 0.59-16.67 | 20a | 5.67-50.98 | 86.67c | 70.32-94.69 |
| 21 | 46.67ac | 24.81-69.89 | 16.67a | 7.34-33.57 | 20a | 5.67-50.98 | 83.33c | 66.43-92.66 |
| 22 | 0a | 0.00-20.39 | 3.33a | 0.59-16.67 | 60b | 31.27-83.18 | 96.67c | 83.33-99.41 |
| 23 | 0a | 0.00-20.39 | 16.67a | 7.34-33.57 | 100b | 72.25-100.00 | 100b | 88.65-100.00 |
| 24 | 26.67a | 10.90-51.95 | 23.33a | 11.79-40.93 | 100b | 72.25-100.00 | 100b | 88.65-100.00 |
| 25 | 0a | 0.00-20.39 | 0a | 0.00-11.35 | 10a | 1.79-40.42 | 100b | 88.65-100.00 |
| 26 | 6.67a | 1.19-29.82 | 10a | 3.46-25.62 | 100b | 72.25-100.00 | 100b | 88.65-100.00 |
| 27 | 13.33ab | 3.73-37.88 | 3.33a | 0.59-16.67 | 40b | 16.82-68.73 | 100c | 88.65-100.00 |
| 28 | 20a | 7.05-45.19 | 16.67a | 7.34-33.57 | 47.37a | 21.79-74.41 | 100b | 88.65-100.00 |
| 29 | 6.67a | 1.19-29.82 | 40a | 24.59-57.68 | 10a | 1.79-40.42 | 100b | 88.65-100.00 |
| 30 | 0a | 0.00-20.39 | 0a | 0.00-11.35 | 100b | 72.25-100.00 | 100b | 88.65-100.00 |
| **Mean** | 22.61 |  | 24.27 |  | 60.02 |  | 89.10 |  |

*Different letters indicate significant differences between the values in the same line (p < 0.05).

** Significant differences were assessed through the overlapping of the 95%CI.
