# Supplementary material for: Cross-sectional study of seropositivity, lung lesions and associated risk factors of the main pathogens of Porcine Respiratory Diseases Complex (PRDC) in Goiás, Brazil
Source: Porcine Health Manag. 2019 Oct 14;5:23. doi: 10.1186/s40813-019-0130-0 (PMC6791015; doi:10.1186/s40813-019-0130-0)
Supplement: Supplementary file 3 — Additional file 3. Actinobacillus pleuropneumoniae seroprevalence in the weaners, growers, finishers and pigs at slaughter from the 30 sampled herds from the state of Goiás, Brazil, and the respective 95% confidence interval (CI 95%). [file 40813_2019_130_MOESM3_ESM.docx]

**Supplementary Document III.** *Actinobacillus pleuropneumoniae* seroprevalence in the weaners, growers, finishers and pigs at slaughter from the 30 sampled herds from the state of Goiás, Brazil, and the respective 95% confidence interval (CI 95%).

| **APP** | **Nursery (n=15)** | | **Growing (n=30)** | | **Finishing (n=10)** | | **Slaughter (n=30)** | |
| --- | --- | --- | --- | --- | --- | --- | --- | --- |
| **Herd ID** | **Prevalence (%)** | **CI 95%** | **Prevalence (%)** | **CI 95%** | **Prevalence**  **(%)** | **CI 95%** | **Prevalence (%)** | **CI 95%** |
| **1** | 13.33a | 3.73-37.88 | 10a | 3.46-25.62 | 10a | 1.79-40.42 | 5a | 1.16-19.05 |
| **2** | 0a | 0.00-20.39 | 0a | 0.00-11.35 | 0a | 0.00-27.75 | 0a | 0.00-11.35 |
| **3** | 0a | 0.00-20.39 | 0a | 0.00-11.35 | 0a | 0.00-27.75 | 0a | 0.00-11.35 |
| **4** | 0a | 0.00-20.39 | 25a | 12.98-42.70 | 0a | 0.00-27.75 | 3.33a | 0.59-16.67 |
| **5** | 0a | 0.00-20.39 | 0a | 0.00-11.35 | 0a | 0.00-27.75 | 0a | 0.00-11.35 |
| **6** | 0a | 0.00-20.39 | 0a | 0.00-11.35 | 0a | 0.00-27.75 | 0a | 0.00-11.35 |
| **7** | 26.67a | 10.90-51.95 | 0a | 0.00-11.35 | 0a | 0.00-27.75 | 10a | 3.46-25.62 |
| **8** | 28.57a | 12.08-53.80 | 0b | 0.00-11.35 | 0ab | 0.00-27.75 | 0b | 0.00-11.35 |
| **9** | 69.23a | 44.10-86.52 | 0b | 0.00-11.35 | 0b | 0.00-27.75 | 6.67b | 1.85-21.33 |
| **10** | 25a | 9.89-50.30 | 5a | 1.16-19.05 | 4.55a | 0.44-33.89 | 0a | 0.00-11.35 |
| **11** | 0a | 0.00-20.39 | 0a | 0.00-11.35 | 0a | 0.00-27.75 | 0a | 0.00-11.35 |
| **12** | 6.67a | 1.19-29.82 | 0a | 0.00-11.35 | 10a | 1.79-40.42 | 0a | 0.00-11.35 |
| **13** | 13.33a | 3.73-37.88 | 0a | 0.00-11.35 | 0a | 0.00-27.75 | 0a | 0.00-11.35 |
| **14** | 0a | 0.00-20.39 | 0a | 0.00-11.35 | 0a | 0.00-27.75 | 6.67a | 1.85-21.33 |
| **15** | 20a | 7.05-45.19 | 0a | 0.00-11.35 | 10a | 1.79-40.42 | 10a | 3.46-25.62 |
| **16** | 20a | 7.05-45.19 | 0a | 0.00-11.35 | 0a | 0.00-27.75 | 0a | 0.00-11.35 |
| **17** | 13.33a | 3.73-37.88 | 0a | 0.00-11.35 | 0a | 0.00-27.75 | 0a | 0.00-11.35 |
| **18** | 80a | 54.81-92.95 | 0b | 0.00-11.35 | 0b | 0.00-27.75 | 33.3b | 19.20-51.19 |
| **19** | 0a | 0.00-20.39 | 0a | 0.00-11.35 | 0a | 0.00-27.75 | 6.67a | 1.85-21.33 |
| **20** | 6.67ab | 1.19-29.82 | 0a | 0.00-11.35 | 0ab | 0.00-27.75 | 43.33b | 27.37-60.80 |
| **21** | 20a | 7.05-45.19 | 0a | 0.00-11.35 | 0a | 0.00-27.75 | 0a | 0.00-11.35 |
| **22** | 60a | 35.75-80.18 | 0b | 0.00-11.35 | 0b | 0.00-27.75 | 10b | 3.46-25.62 |
| **23** | 46.67a | 24.81-69.89 | 0b | 0.00-11.35 | 0b | 0.00-27.75 | 0b | 0.00-11.35 |
| **24** | 13.33a | 3.73-37.88 | 0b | 0.00-11.35 | 0b | 0.00-27.75 | 0b | 0.00-11.35 |
| **25** | 6.67a | 1.19-29.82 | 0a | 0.00-11.35 | 10a | 1.79-40.42 | 16.67a | 7.34-33.57 |
| **26** | 33.33a | 15.17-58.28 | 3.33a | 0.59-16.67 | 0a | 0.00-27.75 | 20a | 9.50-37.31 |
| **27** | 18.75a | 6.38-43.86 | 0a | 0.00-11.35 | 0a | 0.00-27.75 | 13.33a | 5.31-29.68 |
| **28** | 26.67a | 10.90-51.95 | 0a | 0.00-11.35 | 0a | 0.00-27.75 | 0a | 0.00-11.35 |
| **29** | 33.33a | 15.17-58.28 | 0a | 0.00-11.35 | 0a | 0.00-27.75 | 10a | 3.46-25.62 |
| **30** | 40a | 19.82-64.25 | 3.33b | 0.59-16.67 | 0ab | 0.00-27.75 | 0b | 0.00-11.35 |
| **Mean** | 20.72 |  | 1.55 |  | 1.48 |  | 6.49 |  |

*Different letters indicate significant differences between the values in the same line (p < 0.05).

** Significant differences were assessed through the overlapping of the 95%CI.
