# Supplementary material for: Cross-sectional study of seropositivity, lung lesions and associated risk factors of the main pathogens of Porcine Respiratory Diseases Complex (PRDC) in Goiás, Brazil
Source: Porcine Health Manag. 2019 Oct 14;5:23. doi: 10.1186/s40813-019-0130-0 (PMC6791015; doi:10.1186/s40813-019-0130-0)
Supplement: Supplementary file 4 — Additional file 4. SIV seroprevalence in the weaners, growers, finishers and pigs at slaughter from the 30 sampled herds from the state of Goiás, Brazil, and the respective 95% confidence interval (CI 95%). [file 40813_2019_130_MOESM4_ESM.docx]

**Supplementary Document IV.** SIV seroprevalence in the weaners, growers, finishers and pigs at slaughter from the 30 sampled herds from the state of Goiás, Brazil, and the respective 95% confidence interval (CI 95%).

| **SIV** | **Nursery (n=15)** | | **Growing (n=30)** | | **Finishing (n=10)** | | **Slaughter (n=30)** | |
| --- | --- | --- | --- | --- | --- | --- | --- | --- |
| **Herd ID** | **Prevalence (%)** | **CI 95%** | **Prevalence (%)** | **CI 95%** | **Prevalence**  **(%)** | **CI 95%** | **Prevalence (%)** | **CI 95%** |
| 1 | 66.67a | 41.72-84.83 | 6.67b | 1.85-21.33 | 0b | 0.00-27.75 | 0b | 0.00-11.35 |
| 2 | 13.33a | 3.73- 37.88 | 57.14b | 39.64-73.02 | 0a | 0.00-27.75 | 3.33a | 0.59-16.67 |
| 3 | 92.86a | 69.58-98.67 | 83.33a | 66.43-92.66 | 100a | 72.25-100.00 | 100a | 88.65-100.00 |
| 4 | 86.67a | 62.12-96.27 | 63.33ab | 45.51-78.12 | 60ab | 31.27-83.18 | 30b | 16.66-47.88 |
| 5 | 0a | 0.00-20.39 | 0a | 0.00-11.35 | 0a | 0.00-27.75 | 3.33a | 0.59-16.67 |
| 6 | 66.67ac | 41.72-84.83 | 100b | 88.65-100.00 | 50a | 23.66-76.34 | 96.67bc | 83.33-99.41 |
| 7 | 60a | 35.75-80.18 | 96.67b | 83.33-99.41 | 100ab | 72.25-100.00 | 96.67b | 83.33-99.41 |
| 8 | 92.86a | 69.58- 98.67 | 22.22b | 11.02-39.73 | 40bc | 16.82-68.73 | 76.67ac | 59.07-88.21 |
| 9 | 0a | 0.00-20.39 | 0a | 0.00-11.35 | 0a | 0.00-27.75 | 0a | 0.00-11.35 |
| 10 | 46.67a | 24.81-69.89 | 70a | 52.12- 83.34 | 30a | 10.78-60.32 | 100b | 88.65-100.00 |
| 11 | 13.33a | 3.73-37.88 | 86.67b | 70.32-94.69 | 45.45ab | 20.45-72.97 | 46.67a | 30.24-63.86 |
| 12 | 57.14ab | 33.29-78.08 | 40a | 24.59-57.68 | 100bc | 72.25-100.00 | 100c | 88.65-100.00 |
| 13 | 73.33ab | 48.05-89.10 | 56.67a | 39.20-72.63 | 100ab | 72.25-100.00 | 100b | 88.65-100.00 |
| 14 | 26.67a | 10.90-51.95 | 90b | 74.38-96.54 | 70ab | 39.68-89.22 | 76.67b | 59.07-88.21 |
| 15 | 40a | 19.82-64.25 | 96.67b | 83.33-99.41 | 80ab | 49.02-94.33 | 93.33b | 78.67-98.15 |
| 16 | 13.33a | 3.73-37.88 | 100b | 88.65-100.00 | 100b | 72.25-100.00 | 93.33b | 78.67-98.15 |
| 17 | 40a | 19.82-64.25 | 100b | 88.65-100.00 | b100 | 72.25-100.00 | 93.33b | 78.67-98.15 |
| 18 | 13.33a | 3.73-37.88 | 93.33b | 78.67-98.15 | 100b | 72.25-100.00 | 100b | 88.65-100.00 |
| 19 | 46.67a | 24.81-69.89 | 86.67b | 70.32-94.69 | 60ab | 31.27-83.18 | 80ab | 62.69-90.50 |
| 20 | 13.33ab | 3.73-37.88 | 6.67a | 1.85-21.33 | 50bc | 23.66-76.34 | 78.57c | 61.13-89.53 |
| 21 | 13.33a | 3.73-37.88 | 3.33a | 0.59-16.67 | 100b | 72.25-100.00 | 83.33b | 66.43-92.66 |
| 22 | 20a | 7.05-45.19 | 100b | 88.65-100.00 | 100b | 72.25-100.00 | 96.67b | 83.33-99.41 |
| 23 | 53.33a | 30.11-75.19 | 96.67b | 83.33-99.41 | 100b | 72.25-100.00 | 100b | 88.65-100.00 |
| 24 | 66.67a | 41.72-84.83 | 96.67ab | 83.33-99.41 | 100ab | 72.25-100.00 | 100b | 88.65-100.00 |
| 25 | 33.33a | 15.17-58.28 | 100b | 88.65-100.00 | 50a | 23.66-76.34 | 96.67b | 83.33-99.41 |
| 26 | 26.67ac | 10.90-51.95 | 96.67b | 83.33-99.41 | 100bc | 72.25-100.00 | 66.67ac | 48.78-80.77 |
| 27 | 46.67a | 24.81-69.89 | 100b | 88.65-100.00 | 60b | 31.27-83.18 | 83.33ab | 66.43-92.66 |
| 28 | 26.67a | 10.90-51.95 | 100b | 88.65-100.00 | 10a | 1.79-40.42 | 3.33a | 0.59-16.67 |
| 29 | 60a | 35.75-80.18 | 86.67ab | 70.32-94.69 | 80ab | 49.02-94.33 | 100b | 88.65-100.00 |
| 30 | 46.67ac | 24.81-69.89 | 46.67a | 30.24-63.86 | 100bc | 72.25-100.00 | 83.33c | 66.43-92.66 |
| **Mean** | 41.87 |  | 69.42 |  | 66.18 |  | 72.73 |  |

*Different letters indicate significant differences between the values in the same line (p < 0.05).

** Significant differences were assessed through the overlapping of the 95%CI.
